# Fiber-Amplifier-Enhanced QEPAS Sensor for Simultaneous Trace Gas Detection of NH_3_ and H_2_S

**DOI:** 10.3390/s151026743

**Published:** 2015-10-21

**Authors:** Hongpeng Wu, Lei Dong, Xiaoli Liu, Huadan Zheng, Xukun Yin, Weiguang Ma, Lei Zhang, Wangbao Yin, Suotang Jia

**Affiliations:** State Key Laboratory of Quantum Optics and Quantum Optics Devices, Institute of Laser Spectroscopy, Shanxi University, Taiyuan 030006, China; E-Mails: beijing2008whp@163.com (H.W.); liuxiaoli@163.com (X.L.); zhenghuadan@126.com (H.Z.); yinxukun@163.com (X.Y.); mwg@sxu.edu.cn (W.M.); k1226@sxu.edu.cn (L.Z.); ywb65@sxu.edu.cn (W.Y.); tjia@sxu.edu.cn (S.J.)

**Keywords:** quartz enhanced photoacoustic spectroscopy, near-infrared distributed feedback laser, erbium doped fiber amplifier, modulation cancelation method, multi-component traces gas detection

## Abstract

A selective and sensitive quartz enhanced photoacoustic spectroscopy (QEPAS) sensor, employing an erbium-doped fiber amplifier (EDFA), and a distributed feedback (DFB) laser operating at 1582 nm was demonstrated for simultaneous detection of ammonia (NH_3_) and hydrogen sulfide (H_2_S). Two interference-free absorption lines located at 6322.45 cm^−1^ and 6328.88 cm^−1^ for NH_3_ and H_2_S detection, respectively, were identified. The sensor was optimized in terms of current modulation depth for both of the two target gases. An electrical modulation cancellation unit was equipped to suppress the background noise caused by the stray light. An Allan-Werle variance analysis was performed to investigate the long-term performance of the fiber-amplifier-enhanced QEPAS sensor. Benefitting from the high power boosted by the EDFA, a detection sensitivity (1σ) of 52 parts per billion by volume (ppbv) and 17 ppbv for NH_3_ and H_2_S, respectively, were achieved with a 132 s data acquisition time at atmospheric pressure and room temperature.

## 1. Introduction

The use of trace gas detectors is widespread in such diverse fields as in industrial process control, medical diagnostics, atmospheric science and environment monitoring [1,2]. Laser photoacoustic spectroscopy (PAS) is a well-established method, which can be used for detecting or monitoring chemical substances in gases [3,4]. In some cases, quantification of chemical species at part per trillion (ppt) concentration levels can be achieved by use of PAS [5]. The sensitivity of the detecting element, for example a microphone, and the geometry of the photoacoustic cell determine the detection sensitivity of the PAS-based sensors. However, most of the photoacoustic cells have a resonance at low frequency values (<2 kHz), which makes them more sensitive to environmental noise [6]. Moreover, their size and weight are considered to be large and heavy in field applications [7,8,9]. Quartz-enhanced photoacoustic spectroscopy (QEPAS) technique, first reported in 2002 [10], is a recent modification of conventional PAS, in which a low cost (<$1) commercially available quartz tuning fork (QTF) acts as an acoustic wave transducer to detect the sound signal generated by the trace gas absorbing the excitation laser beam [10,11]. The QTF’s high resonant frequency of ~32.768 KHz and *Q*-factor of ~12,000 (in atmosphere) improves QEPAS selectivity and immunity to environmental acoustic noise. In addition, the ~2 mm^3^ dimensions of the QTF results in an ultra-compact gas cell and a fast gas exchange [12]. 

A distinct advantage of QEPAS technique inherited from traditional PAS is the excitation-wavelength independence [13]. This benefit allows the same QEPAS-based trace gas sensor to be used with any type of laser (e.g., distributed feedback (DFB) laser [14], quantum cascade laser (QCL) laser [15] and light emitting diode (LED) [16,17]) and any wavelength (e. g., visible [16], near-infrared (NIR) [18], mid-infrared (MIR) [19] and THz spectral region [20,21,22]). As a result, multi-gas detection is achievable by use of a single QEPAS module [8,12,23]. Efficient multi-gas QEPAS sensors have been demonstrated with the same [12,23] and different [8,13] excitation sources. 

The excellent linear relationship between the sensitivity and incident laser power provided by the QTF is another outstanding feature of a QEPAS-based detector [8], which makes the performance of QEPAS based sensor be able to benefit from the enhanced excitation laser power. Especially when the QEPAS detection was performed in NIR region, boosting laser power is an effective way to compensate the line strengths of the weak vibrational overtones. An optical fiber amplifier is an excellent choice for boosting laser power, which can achieve amplification factors of up to three orders of magnitude for input power without obvious line-width broadening [24]. Thanks to the development of the telecommunications industry, the fiber amplifiers are currently able to efficiently work in S band (1450–1550 nm), C band (1520–1570 nm) and L band (1565–1610 nm). However, the fiber amplifiers are receiving more attentions in C band for gas sensing, as several species, such as NH_3_, CO, CO_2_, HCN, H_2_S and C_2_H_2_, have spectra within this range. 

In this paper, we describe a QEPAS based sensor system for detecting the ammonia (NH_3_) and hydrogen sulfide (H_2_S) simultaneously. NH_3_ is a toxic, reactive and corrosive gas, which is extensively used to manufacture fertilizers, explosives and pharmaceuticals [25]. It plays an important role in commercial refrigerants and is an important pollution tracer gas for the terrestrial atmosphere [26]. H_2_S is a colorless, toxic, flammable gas. It has an important impact on the field of chemical industry and atmospheric chemistry [27,28]. And, even at low concentration levels, H_2_S is dangerous to human life [29]. In addition, NH_3_ and H_2_S appear together at many applications. Detecting NH_3_ and H_2_S simultaneously is relevant in the field of human pathologies, renewable energies, environmental monitoring, *etc.* For example, doctors can diagnose a disease such as bacterial overgrowth of the small intestine or peptic ulcer disease by analyzing the content of H_2_S and NH_3_ in human breath. Hence, there is a need for the development of reliable, cost-effective trace gas sensors, capable of detecting NH_3_ and H_2_S simultaneously for applications in human pathologies, renewable energies, *etc.* [30,31]. Recently some QEPAS-based NH_3_ and H_2_S sensors have been reported. Kosterev *et al.* [25], Serebryakov *et al.* [32] and Dong *et al.* [33] reported the QEPAS-based NH_3_ detector using a NIR laser source. Viciani *et al.* [34], Sicicliani de Cumis *et al.* [19] and Spagnolo *et al.* [20] investigated the QEPAS-based H_2_S sensors with a NIR, MIR and THz laser sources, respectively. However, a sensor, capable of detecting NH_3_ and H_2_S simultaneously has not been reported. In addition, the sensor systems described in these papers were operated at lower pressure to improve the detecting limit, which increases the sensor size since a pressure control system, consisting of a vacuum pump, a pressure controller, valves, *etc.*, has to be added. In the following section, we report the development of a fiber-amplifier-enhanced QEPAS sensor for the simultaneous dual-species monitoring using a single NIR DFB laser with an output wavelength of 1582 nm. This approach combines a watt-level excitation laser source and QEPAS acoustic detection module (ADM), which takes advantage of QEPAS linear relationship between the sensitivity and incident laser power, offering a low-cost, highly sensitive, reliable QEPAS sensor for dual-species detection.

## 2. Experimental Setup

**Figure 1:**
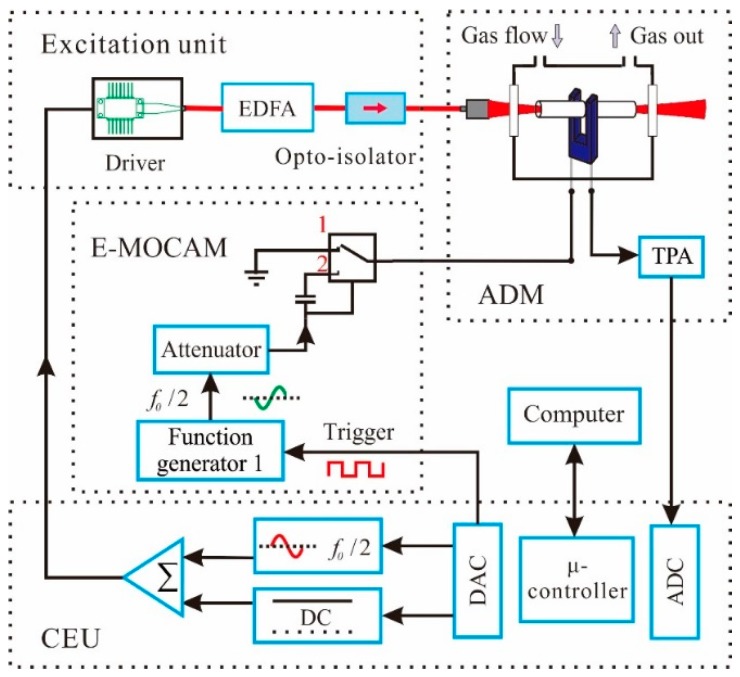
Schematic drawing of the experimental setup. EDFA: erbium-doped fiber amplifier; TPA: transimpedance preamplifier; ADM: acoustic detection module; E-MOCAM: electrical modulation cancellation unit; CEU: control electronics unit.

For sensitive NH_3_ and H_2_S concentration measurements, a wavelength modulation spectroscopy (WMS) with 2nd harmonic detection was utilized [35,36]. The following measurements were performed with the setup shown schematically in Figure 1. It consists of a control electronics unit (CEU), an excitation unit, acoustic detection module (ADM), and electrical modulation cancellation method (E-MOCAM) unit. The CEU performed the functions of measuring the QTF parameters (resonant frequency *f*_0_ ~ 32,762 Hz, *Q* factor ~ 11,800 and resistance *R* ~ 150 kΩ), modulating the laser current at half of the QTF resonance frequency (*f* = *f*_0_/2) and locking the laser wavelength to the target absorption line, as well as measuring the current generated by the QTF in response to the photoacoustic signal. The excitation unit contains a NIR DFB diode laser (FITEL Inc. (Tokyo, Japan), Model FRL15DCWD-A82) with a center wavelength of 1582.1 nm, an erbium-doped fiber amplifier (Connect Laser Technology Ltd. (Shanghai, China), Model MFAS-L-EY-B-MP) and an opto-isolator (Connect Laser Technology Ltd. Model A12104132). The DFB laser was mounted onto a driver board, which was used to control the laser temperature and current. The EDFA offers an adjustable output power in the range of 30 mW to 1500 mW with the same wavelength as the seed laser. The opto-isolator was utilized to protect the high power laser against back reflections. The output laser beam from the opto-isolator was directed to a fiber focuser (L-com Inc. (North Andover, MA, USA), Model 163429-01) with a beam output of 0.1 mm-diameter light spot, and then passed through the ADM which includes a QTF, an acoustic micro-resonator (AmR) and a low noise transimpedance preamplifier (TPA) with a feedback resistor of *R_g_ =* 10 MΩ. The AmR, including two identical metallic tine tubes of 4.0 mm in length with a 0.8 mm inner diameter and a 1.24 mm outer diameter, was equipped in an “on beam” configuration. Compared with the “off beam” configuration employed in previous works in [18], the “on beam” configuration can further enhance the QEPAS signal [37,38]. Specifically, the tubes were placed along the excitation laser beam, below the QTF opening 0.7 mm, and close to QTF with a 30 μm gap. The QTF and AmR were enclosed inside a gas enclosure, filled with the trace gas of a fixed concentration, with two antireflection-coated CaF_2_ windows. The E-MOCAM unit was equipped to suppress the background noise caused by stray light. This method has been reported in our previous article in detail [18]. 

**Figure 2:**
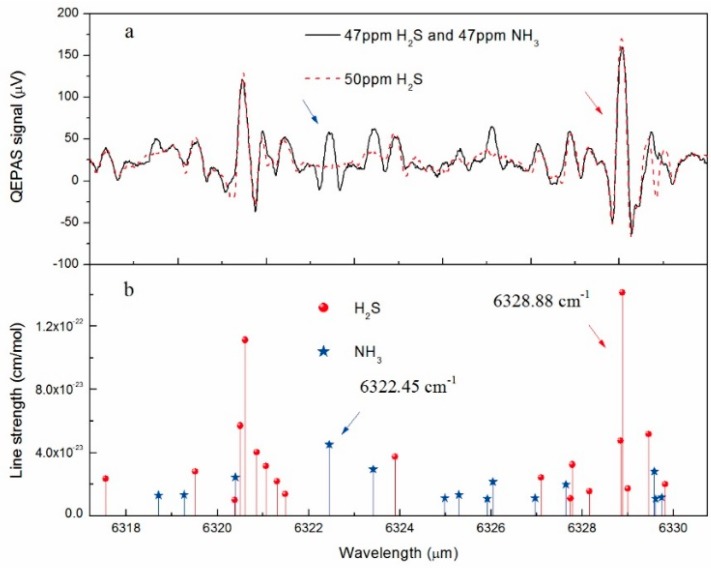
(**a**): 2*f* QEPAS signal when the laser temperature is scanned with 1250 mW laser power at atmospheric pressure and room temperature. The spectrum plotted by a black line is from 47 ppm H_2_S:47 ppm NH_3_:N_2_; the spectrum plotted by a red dash line is from 50 ppm H_2_S:N_2_; (**b**): Line strengths of the H_2_S and NH_3_ transitions, as reported in the HITRAN database, within the frequency span of Figure 2a.

The absorption line selection for NH_3_ and H_2_S detection was first performed. The 2*f* wavelength-modulation spectroscopy (WMS) QEPAS signal of two different measurements was recorded in the emitted wavelength range of the available DFB laser, 6317.2–6330.75 cm^−1^, by varying the temperature of the laser from 40 °C to 5 °C in steps of 0.05 degree via CEU. The driving current of the excitation laser for generating the acoustic wave was set at 160 mA with a modulation depth of ~20 mA. The signal output from one electrode of the QTF was directed to CEU, for 300 ms lock-in integration time, after the current signal was converted into a voltage signal via a custom TPA. Meanwhile, the other electrode was connected to the ground (the switch of the E-MOCAM unit in the Figure 1 was set to position 1). The amplifier was set to a 12 dB/oct filter slope. The spectrum plotted by a black line in the top of Figure 2 was obtained when the gas cell was filled with 47 ppm H_2_S: 47 ppm NH_3_:N_2_ mixture at atmospheric pressure (P = 760 Torr) and room temperature (T = 297.2 K) with an output power of 1250 mW, and the spectrum plotted by a red dash line was the result of scanning 50 ppm H_2_S:N_2_ mixture at the same condition. The plot in the bottom of Figure 2 is a representation of the intensities and positions of the same absorption lines from the HITRAN database [39]. The relative position and intensities of the absorption lines are in good agreement with the HITRAN data. For accurate QEPAS measurements, interference free NH_3_ and H_2_S rotational-vibration absorption lines located at 6322.45 cm^−1^ and 6328.88 cm^−1^ were selected, respectively, which have the strongest line intensity available for the two gases in the exciting laser operating range. In addition, the selected target line of the H_2_S merges with a weak neighbor line located at 6328.84 cm^−1^, thus resulting in slight asymmetry of the QEPAS signal.

A crucial issue for NH_3_ and H_2_S measurements is to avoid interference effects from H_2_O, as the presence of H_2_O not only affects the H_2_S QEPAS signal amplitude via acting as a promoter of vibrational-translational relaxation process, but also changes or even destroys the H_2_S and NH_3_ spectrum if the target line is close to a H_2_O absorption line with a similar line strength. Fortunately, the strength of water absorption lines, lying in the wavelength range of the emitting laser, is lower than 1 × 10^−25^ cm/mol, which is ~1000 times weaker than the strength of the target lines. Therefore, the presence of water vapor does not sensibly affect the NH_3_ and H_2_S absorption profile. 

To simultaneously detect NH_3_ and H_2_S, two sets of CEU parameters were pre-programmed and switched automatically by CEU at the user-set interval, as described in [33]. The laser current and temperature, modulation depth, switch time, and regulation parameters corresponding to the two target gases respectively, are included in each set. The parameters were optimized via experiments as mentioned below. 

## 3. Results and Discussion

### 3.1. Analysis of Saturation Effect and Optimization of Modulation Depth

**Figure 3:**
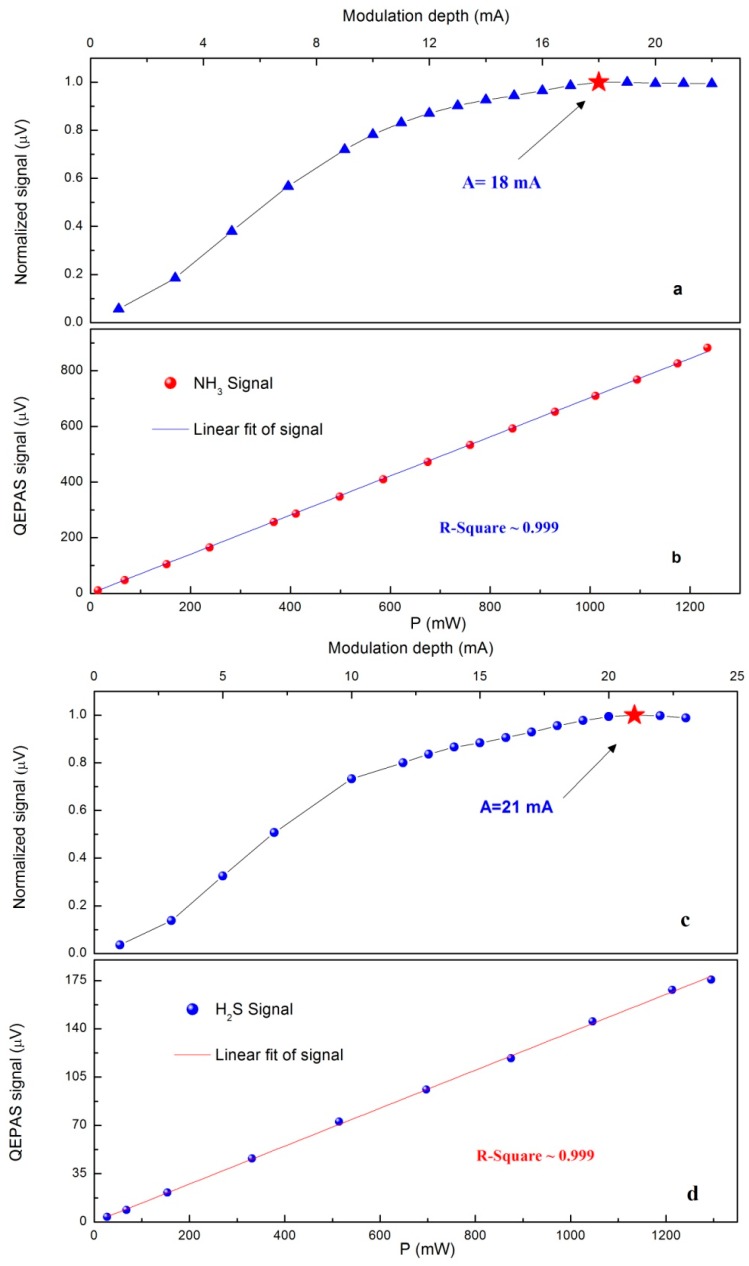
(**a**,**c**): The normalized signal amplitude as a function of the current modulation depth for NH_3_ and H_2_S, respectively; (**b**,**d**): Fiber-amplifier-enhanced QEPAS signal as a function of the actual laser power for NH_3_ and H_2_S, respectively.

With a low optical excitation power, the detected signal *S* of the QEPAS sensor can be expressed as [18]:

(1) S=k⋅P⋅C⋅α⋅ε⋅Q

where *k* is a constant describing system parameters, *P* is the incident optical power, *C* is the detected gas concentration, *α* is the peak intensity of 2*f* absorption spectrum, *ε* is the conversion efficiency of the absorbed optical radiation power into acoustic energy, and *Q* is the quality factor of QTF. *α*, *ε* and *Q* are pressure dependent. In addition, *α* depends on the modulation depth of the laser current. When the modulation width is close to the absorption linewidth, the maximum 2*f* signal is achieved. Therefore, in order to optimize the sensor performance, both the gas pressure and the WM depth must be appropriately selected. However, the fiber-amplifier-enhanced QEPAS sensor was operated at atmospheric pressure to avoid using the pressure controller and the powerful vacuum pump. Only the modulation depth for NH_3_ and H_2_S were optimized by experiments. These optimizations were carried out with 1000 ppm NH_3_:N_2_ and 50 ppm H_2_S:N_2_, respectively, and the detections were based on 2*f*-WMS approach by dithering and scanning the laser current. The absorption lines of NH_3_ and H_2_S at 6322.45 cm^−1^ and 6328.88 cm^−1^, respectively, were employed. As shown in Figure 3a,c, the optimized modulation depth were ~18 mA for NH_3_ channel and ~21 mA for H_2_S channel at atmospheric press. 

In Equation (1), the signal amplitude of the QEPAS-based sensor is proportional to incident laser power. However, when the excitation optical power is sufficiently high, saturation effects may result in invalidation of the Equation (1) [40,41]. An experiment was carried out to detect the QEPAS 2*f* signal amplitudes of NH_3_ and H_2_S channels at atmospheric pressure and room temperature, as a function of the excitation powers. The results were plotted in Figure 3b,d with linear fittings. The excellent linearity of the fiber-amplifier-enhanced QEPAS sensor in response to the laser power confirms that saturation does not occur. In order to obtain the maximum signal, further evaluation tests in this paper were performed with ~1250 mW incident laser power. 

### 3.2. Background Noise Elimination and Performance Evaluation of the Fiber-Amplifier-Enhanced QEPAS Sensor

**Figure 4:**
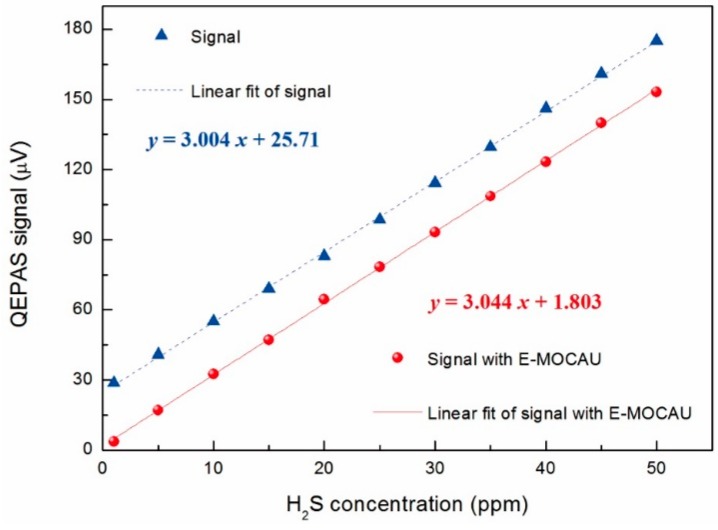
Fiber-amplifier-enhanced QEPAS signal as a function of different H_2_S concentrations. Triangle and dash line, fiber-amplifier-enhanced QEPAS signal without E-MOCAM; Circle and solid line, fiber-amplifier-enhanced QEPAS signal with E-MOCAM.

To verify the linear response of the H_2_S channel with respect to different concentrations, a calibration mixture of 50 ppm H_2_S:N_2_ was diluted with dry N_2_ to obtain different concentration levels. For each concentration level, continuous monitoring with 1 s data acquisition time was performed. The laser frequency was locked to the center of the targeted absorption line (6328.88 cm^−1^). The data averaged and plotted as a function of H_2_S concentrations are shown in Figure 4. A good linear dependence of the fiber-amplifier-enhanced QEPAS signal on the H_2_S concentrations was observed. However, the linear fit resulted in a noise floor of ~26 μV, as shown by the dash line in Figure 4. 

Several experimental studies showed that the theoretical noise of the traditional QEPAS-based sensor is equal to the thermal noise of the QTF and the feedback resistor [26]:

(2) $$\sqrt{V_{N-R}^{2}}+\sqrt{V_{N-R_{g}}^{2}}=\frac{R_{g}}{\sqrt{2}}\sqrt{\frac{4k_{B}T\Delta f}{R}}+\frac{\sqrt{4k_{B}TR_{g}\Delta f}}{\sqrt{2}}$$

(3) $$R=\frac{1}{Q}\sqrt{\frac{L}{C}}$$

where *k_B_* is the Boltzmann constant (*k_B_* = 1.38 × 10^−23^ J/K), *T* is QTF temperature (T = 297.2 K), Δ*f* is the detection bandwidth (Δ*f* = 0.833 Hz), *R =* 648 kΩ, *L* = 6038 H and *C =* 3.9 × 10^−15^ F are the electrical parameters of the QTF when it is represented by an equivalent series resonant circuit. Hence, the theoretical thermal noise value, ~1.5 μV, is ~18 times less than the experimental background floor noise. The offset noise floor is mainly caused by the stray light from the EDFA. The power of the stray light was absorbed by the QTF and the AmR as well as the CaF_2_ windows, and subsequently converted into the extra noise. To remove the offset noise floor, an electrical modulation cancellation method (E-MOCAM) was used [16,18,42,43], as shown in the dotted box in Figure 1. In this case, the switch in the E-MOCAM unit was set to position 2 to exert a sine wave, with the same frequency *f*_0_ as the QTF resonant frequency but the opposite phase, on the QTF via its electrode. The amplitude of the sine wave, generated by a function generator (Agilent Model 33210A), was attenuated by an electrical attenuator before it was sent to the QTF to balance the noise floor. 

**Figure 5:**
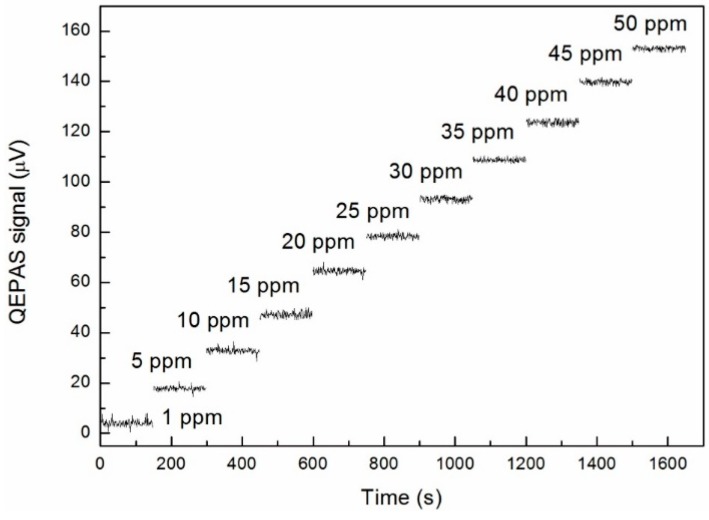
Fiber-amplifier-enhanced QEPAS signal repetitively recorded as a function of time for H_2_S concentration values ranging from 1 ppm to 50 ppm.

The linearity of this sensor’s H_2_S channel was reevaluated by measuring its response to the different H_2_S concentrations. The results are shown in Figure 5. The same data are averaged and plotted in Figure 4 using red filled circles. The virtually identical slope of the two fitting lines, before and after equipping the E-MOCAM unit, and the eventual small noise floor level of ~1.803 μV indicates the E-MOCAM well eliminated the background noise. The noise level (1σ) based on scatter data in Figure 5 was ~1.65 μV, which is in agreement with the thermal noise. It results in a noise-equivalent concentration (NEC) of ~535 ppb with a 1 s averaging time and 1250 mW excitation laser power, which corresponds to a normalized noise equivalent absorption coefficient (NNEA) of 1.395 × 10^−9^ W·cm^−1^/√Hz. The higher sensitivity was obtained with the sensor in this paper, compared with the result in [18], in which the NEC and NNEA of the H_2_S sensor were 734 ppb and 9.8 × 10^–9^ W·cm^−1^/√Hz, respectively, though the excitation power in this paper is 150 mW lower. This is due to the stronger line intensity as well as the higher sensitivity enhancement factor for the “on beam” configuration than that of the “off beam” design.

**Figure 6:**
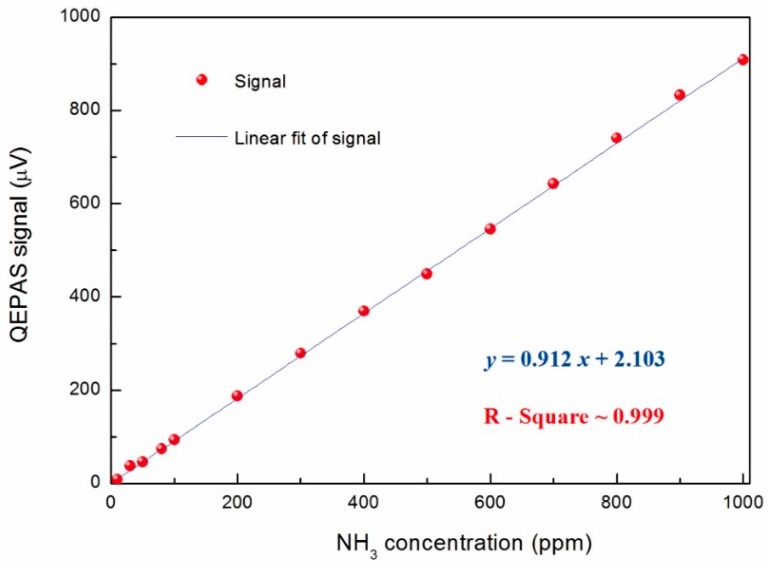
Fiber-amplifier-enhanced QEPAS signal as a function of different NH_3_ concentrations. The data points were the average value in different NH_3_ concentration repetitively recorded ranging from 10 ppm to 1000 ppm.

Similar measurements were carried out for the NH_3_ channel by locking the laser wavelength at 6322.45 cm^−1^. All of the other experiment parameters were the same as the H_2_S test. Based on the measured results, as shown in Figure 6, a minimum detectable concentration limit of ~1.6 ppm was deduced at 1 s integration time. The corresponding NNEA coefficient was found to be 1.5256 × 10^−9^ W·cm^−1^/√Hz.

**Figure 7:**
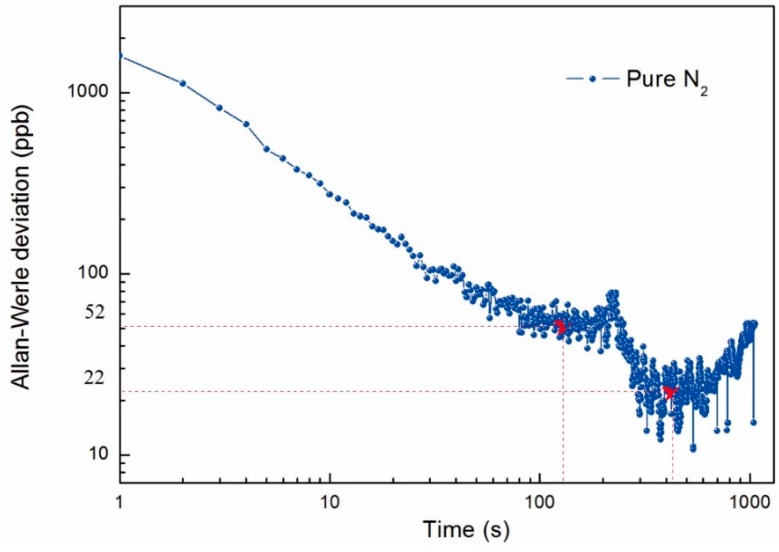
Allan-Werle deviation of the fiber-amplifier-enhanced QEPAS signal as a function of acquisition time. The data were acquired by locking the wavelength at 6322.45 cm^−1^ NH_3_ absorption line with 1 s acquisition time.

To evaluate the long-term stability and precision of such a fiber-amplifier-enhanced QEPAS sensor, an Allen-Werle deviation analysis was performed when pure N_2_ was filled with the ADM at atmospheric pressure and room temperature, and the laser frequency was locked to the NH_3_ absorption line. From the Allen-Werle deviation plot shown in Figure 7, the optimum averaging time for NH_3_ detection is found to be ~132 s, which results in a NEC of ~52 ppb. Since the same equipment and carrier gas are used for H_2_S detection, the H_2_S exhibited the same stability period as the NH_3_ [33]. And the detection limit can be further decreased to ~17 ppb for H_2_S with an integration time of 132 s. 

## 4. Conclusions

A fiber-amplifier-enhanced QEPAS sensor was developed by use of a 1582 nm DFB laser and an EDFA. Two interference-free absorption lines, located at 6328.88 cm^−1^ for H_2_S and 6322.45 cm^−1^ for NH_3_, were identified as the target lines. E-MOCAM and wavelength modulation techniques were employed to reduce the sensor background noise. Based on the linear relationship between the QEPAS sensitivity and incident laser power, this sensor can benefit from a watt-level excitation laser source provided by a commercially available EDFA. In addition, the sensor was operated at atmospheric pressure, which significantly simplifies the sensor configuration and reduces the cost. After the offset of the sensor floor noise caused by stray light was removed by the E-MOCAM, the linearity of the compact QEPAS sensor for NH_3_ and H_2_S were verified by measuring different concentrations of the two target gases, respectively. From the performed Allan-Werle deviation analysis, the optimum average time of the QEPAS sensor signal is 132 s In this case, a detection limit of 52 ppb and 17 ppb for NH_3_ and H_2_S in N_2_ at atmospheric pressure was achieved, respectively. The advantages of this reported fiber-amplifier-enhanced QEPAS sensor, such as compact size, inexpensive cost as well as high detection sensitivity, make it suitable for applications in human pathologies, industrial chemical, renewable energies, and atmospheric chemistry. 
